# Congenital toxoplasmosis: Should we still care about screening?

**DOI:** 10.1016/j.fawpar.2022.e00162

**Published:** 2022-05-11

**Authors:** Eskild Petersen, Valeria Meroni, Daniel V. Vasconcelos-Santos, Laurent Mandelbrot, Francois Peyron

**Affiliations:** a Institute for Clinical Medicine, Faculty of Health Science, University of Aarhus, Denmark; b European Society for Clinical Microbiology and Infectious Diseases, Emerging Infections Task Force, Basel, Switzerland; c Molecular Medicine Department, University of Pavia, Pavia, Italy; d Universidade Federal de Minas Gerais, Faculdade de Medicina, Belo Horizonte, Minas Gerais, Brazil; e Assistance Publique-Hôpitaux de Paris, Service de Gynécologie-Obstétrique, Hôpital Louis Mourier, Colombes, France; Université de Paris; Inserm IAME-U1137, Paris, France; FHU PREMA, Paris, France; f Institut de Parasitologie et de Mycologie Médicale Hôpital de la Croix Rousse, Lyon, France

**Keywords:** Toxoplasma gondii, Congenital toxoplasmosis, Screening

## Abstract

Prenatal systematic screening for congenital toxoplasmosis has been performed in Austria and France since 1975 and neonatal screening for congenital toxoplasmosis has been part of the New England Newborn screening program since 1986.

In this narrative review we review the data leading up to the systematic screening programs in Austria and France, highlighting the main finding of the European Union funded research in the 1990s and early 2000s. Different descriptive studies of the effect of pre- or postnatal treatment are discussed. *Toxoplasma gondii* has different genetic lineages with different pathogenicity in humans. This means that results in areas with a low pathogenic lineage cannot be extrapolated to an area with highly pathogenic lineages. The importance of meat as a source of infection is discussed in the light of an increased prevalence of *T.gondii* in organic livestock production .

## 1 The pre-screening era

*Toxoplasma gondii* was first characterized as an agent of animal disease. In 1908, Alphonso Splendore observed an arch-shaped parasite infecting rabbits in Brazil (named *Toxoplasma cuniculi)* and C. Nicolle and L. Manceaux concomitantly described the same parasite in the rodent *Ctenodactylis gondi*; resemblance with Leishamania spp. led them to initially name it *Leishmania gondii,* in 1908, and subsequently *Toxoplasma gondii,* in 1909 (Ferguson, 2009).

The first human case ascribed to infection with *T. gondii* was a child with hydrocephalus reported by Jankû in 1923 (Janku, 1923) and Sabin reported the first case of encephalitis due to *T. gondii* (Sabin, 1941). During the 1940s, there was an improved understanding of the cause of maternal infection for congenital toxoplasmosis in newborns. In 1953, Feldman reported a series of 103 children, 99% of whom had eye lesions, 63% had intracranial calcifications, and 56% had psychomotor retardation (Feldman, 1953).”This initiated interest in congenital infection among scientists in Europe (Couvreur, 1955).

In Gothenburg, Sweden, 50% of mothers had had previous infection with *T. gondii* and 2 out of 23,260 children had clinical toxoplasmosis during a 1948–51 study period (Holmdahl and Holmdahl, 1955). A study from Austria reported frequent symptoms in children with congenital toxoplasmosis (Eichenwald, 1957). A French study concluded that treatment prevented transmission from mother to child and reduced the clinical symptoms in children (Couvreur and Desmonts, 1962). A later study from France found that the seroprevalence in pregnant women in Paris was 85%, and there was a high risk of *T.gondii* infection in seronegatives (Desmonts et al., 1965; Desmonts and Couvreur, 1974).

The risk factors for infections are consumption of raw and undercooked meat, exposure to fresh water, soil and fresh produce contaminated with oocysts. Studies over the past twenty years have shown that different genotypes have different pathogenicity with the most pathogenic types found in South America and Africa (Shwab et al., 2014).

Primary prevention of toxoplasmosis is based on relatively simple hygiene measures (Jones and Dubey, 2012).

## 2 Screening programs

### 2.1 France, Austria and Slovenia

Systematic prenatal screening programs were introduced in France and Austria in 1975 (Aspöck and Pollak, 1992; Thulliez, 1992). The Austrian program is based on three tests during pregnancy and the French program involves monthly tests of seronegative pregnant women. Slovenia introduced screening for congenital toxoplasmosis adopting the Austrian model with three serological tests during pregnancy (Logar et al., 2002).

### 2.2 Massachusetts, United States

Since January 1986, in Massachusetts and New Hampshire, newborn screening has been implemented for neonatal screening for congenital toxoplasmosis through an IgM capture immunoassay of blood specimens routinely collected just after birth for metabolic disorders (Guerina et al., 1994). This program is still ongoing.

### 2.3 Denmark

Denmark initiated a neonatal screening program in 1999. The program tested for *Toxoplasma*-specific IgG and IgM eluted from the filter paper obtained day four after birth for metabolic screening. The program was closed in 2007 primarily due to lack of sufficient evidence that neonatal treatment prevented long term recurrent retinochoroiditis (Röser et al., 2010).

### 2.4 Brazil

In addition to recommendations on screening for toxoplasmosis in pregnancy, nationwide neonatal screening for toxoplasmosis Is now to be implemented in each state, after specific national legislation was finally passed in 2020. Particularly in the state of Minas Gerais, where prior experience with population-based neonatal screening disclosed high prevalence and severity of congenital toxoplasmosis (Vasconcelos-Santos et al., 2009), prenatal and neonatal screening program started in early 2022.

## 3 The European multicenter studies 1993–2004

A meeting was held at Staten Serum Institute, Copenhagen, in 1991 to discuss why some countries in Europe performed systematic screening for congenital toxoplasmosis, CT, while others did not recognise CT as a major public health problem (Lebech and Petersen, 1992). Treatment in countries performing screening was offered with spiramycin early in pregnancy and later (often after the 18th gestational week) with a combination of sulfadiazine and pyrimethamine. The key research questions that arose were whether the screening programs prevented mother to child transmission by early treatment, whether treatment prevented sequelae in newborns including hydrocephalus and whether treatment during pregnancy prevented post-natal, late onset retinochoroiditis.

This led to the foundation of a European multicenter consortium, the European Research Network on Congenital Toxoplasmosis, which performed a series of studies on diagnosis, management and screening for infection with *Toxoplasma gondii* in pregnant women and newborn children, supported by three consecutive grants from the 10.13039/501100000780European Union.

Here we briefly describe the main results.

A study on maternal-foetal transmission rates found that the overall maternal-foetal transmission was 29% (95% CI 25–33), which masked a sharp increase in risk according to gestational age from 6% at 13 weeks to 72% at 36 weeks. Foetuses infected in early pregnancy were much more likely to show clinical signs of systemic infection. These effects counterbalance, and women who seroconverted at 24–30 weeks of gestation carried the highest risk (10%) of having a congenitally infected child with early clinical signs who was thus at risk of long-term complications (Dunn et al., 1999).

A study from Lyon, France, followed a cohort of 554 *T. gondii* infected pregnant women from 1987 and 1995. The study compared treatment within 4 weeks after seroconversions with treatment after a delay of 4–7 weeks from seroconversion. The adjusted odds ratios (OR) for mother to child transmission after a treatment delay of 4–7 weeks was 1.29 (95% CI: 0.61, 2.73) and after more than 8 weeks 1.44 (95% CI: 0.60, 3.31). The adjusted OR associated with spiramycin alone compared with pyrimethamine-sulfadiazine treatment was 0.91 (95% CI: 0.45, 1.84) and the OR for no treatment compared with pyrimethamine-sulfadiazine treatment was 1.06 (95% CI: 0.37, 3.03). The authors hypothesized that the absence of an effect of prenatal treatment was due to transmission before the start of treatment (Gilbert et al., 2001).

In a multicenter study of 1208 women with *T.gondii* infection during pregnancy identified by prenatal screening, 72% were first prescribed spiramycin, 19% pyrimethamine-sulphonamide and 9% (mostly infected during the last trimester) were untreated. The odds ratios for mother to child transmission for all women treated after a delay of four to seven weeks was 0.77 (95% CI 0.34–1.69), and after eight weeks or more was 1.33 (0.56–2.89) compared with less than four weeks. The odds ratio per week of treatment delay was 1.01 (0.93–1.08). There was no evidence that transmission risk differed in women first treated with pyrimethamine-sulphonamide versus spiramycin: odds ratio 1.10 (0.63–1.91) or in untreated versus treated women: odds ratio 0.57 (0.27–1.17). The study was unable to demonstrate a beneficial effect of the timing or type of prenatal treatment on the risk of mother to child transmission but a clinically important effect could not be excluded (Gilbert, 2003).

Another study looked at parent reported child development in children aged three to four years with congenital toxoplasmosis identified by prenatal screening and treated during infancy. The study found that in this European setting these children had no risks of abnormal development and behavior similar to uninfected children (Freeman et al., 2005).

The SYROCOT (Systematic Review on Congenital Toxoplasmosis) study included 1438 treated mothers identified by prenatal screening for *T.gondii* infection. The study found that treatment started within 3 weeks of seroconversion reduced mother-to-child transmission compared with treatment started after 8 or more weeks (adjusted odds ratio [OR] 0·48, 95% CI 0·28–0·80; *p* = 0·05) (SYROCOT et al., 2007).

In a study of 550 infected liveborn infants identified by prenatal or neonatal screening and followed up, found no evidence that prenatal treatment significantly reduced the risk of clinical manifestations (adjusted OR for treated vs not treated 1.11, 95% CI 0.61–2.02). Increasing gestational age at seroconversion was strongly associated with increased risk of mother-to-child transmission (OR 1.15, 95% CI 1.12–1.17) and decreased risk of intracranial lesions (0.91, 0.87–0.95), but not with eye lesions (0.97, 0.93–1.00) (SYROCOT et al., 2007).

A study including 281 of 284 infected children who underwent ophthalmic examinations and followed up to a median age of 4.8 years found that one in six children (49/281; 17%) had at least one retinochoroidal lesion, two-thirds of whom (32/49; 65%) had a lesion at the posterior pole and 41% (20/49) had bilateral lesions whether clinical manifestations were present or not at birth. A study of children with retinochoroiditis who had visual acuity measured after 3 years of age, 94% (31/33) had normal vision in the best eye (6/12 Snellen or better), as did 91% of those with a posterior pole lesion (21/23) (Tan et al., 2007).

Another study of the presence of extra-ocular clinical manifestations of congenital toxoplasmosis before (on ultrasound examination) or at birth strongly predicted retinochoroiditis. For instance, all 5 children with intracranial abnormalities on fetal ultrasound developed retinochoroiditis. In addition, the risk of a first retinochoroidal lesion by 4 years of age was 80% for children with severe neurologic sequelae, 39% for children with intracranial lesions and no significant neurologic impairment, and 44% for those with lymphadenopathy or hepatosplenomegaly, when compared to 12% in children with no clinical manifestations. For 92% (230 of 249) of children with no retinochoroiditis detected before 4 months of age, the probability of retinochoroiditis by 4 years was low, 8.0%, whether clinical manifestations were present or not. The study concluded that prenatal treatment did not significantly reduce the risk of retinochoroiditis in this European cohort (Freeman et al., 2008).

The last study published by the consortium looked at serious neurological sequelae disease (SNSD). Prenatal treatment reduced the risk of SNSD and the odds ratio for prenatal treatment, adjusted for gestational age at maternal seroconversion, was 0.24 (95% Bayesian credible intervals 0.07–0.71). This effect was robust to most sensitivity analyses. The number of infected foetuses needed to be treated to prevent one case of SNSD was three (95% Bayesian credible intervals 2–15) after maternal seroconversion at 10 weeks, and 18 (9–75) at 30 weeks of gestation. Pyrimethamine-sulphonamide treatment did not reduce SNSD compared with spiramycin alone (adjusted odds ratio 0.78, 0.21–2.95). The proportion of live-born infants with intracranial lesions detected postnatally who developed SNSD was 31.0% (17.0%–38.1%) (Cortina-Borja et al., 2010).

## 4 The situation after the end of the European multicenter studies

Several additional findings have been reported. In an Austrian registry of 1269 pregnancies (Prusa et al., 2015), the rate of transmission of *T. gondii* after seroconversion was lower after treatment with pyrimethamine + sulfadiazine than after other types of treatment or lack of treatment. This retrospective study accounted for gestational age, although dating of maternal infection was not always precise. A retrospective study in Italy (Valentini et al., 2015) compared mother-to-child transmission rates after maternal primary infection between three different treatment regimens, adjusting for the trimester of infection. The transmission rates were lower in women who received cotrimoxazole + spiramycin or pyrimethamine + sulfonamide than in those receiving spiramycin alone.

A study from France, “TOXOGEST” compared the efficacy and tolerance of pyrimethamine + sulfadiazine vs spiramycin to reduce placental transmission. This was a randomized, open-label trial in 36 French centers, comparing pyrimethamine (50 mg qd) + sulfadiazine (1 g tid) with folinic acid vs spiramycin (1 g tid) following toxoplasmosis seroconversion. In all, 143 women were randomized into specific treatment arms from November 2010 through January 2014. There was a trend toward lower transmission with prenatal treatment with pyrimethamine + sulfadiazine compared to spiramycin (18.5% vs 30%, respectively, *p* = 0.147, Odds ratio = 0.53; 95% CI 0.23–1), but it did not reach statistical significance, possibly for lack of statistical power due to a lower than expected sample size when the study was discontinued (Mandelbrot et al., 2018). To our knowledge, no further randomized studies are planned or underway. The evidence on the impact of prophylactic treatment after maternal toxoplasmosis was reviewed recently (Mandelbrot, 2020).

There are also indirect arguments in favor of early treatment of congenital toxoplasmosis prior to birth. One is the EMSCOT study mentioned above (Cortina-Borja et al., 2010). Other retrospective studies (Hohlfeld et al., 1989; Foulon et al., 1999; Berrébi et al., 2010) found that treatment started prenatally with pyrimethamine + sulfonamides was associated with a reduction of more than two-thirds of the sequelae in infected infants. Kieffer et al. (2008) reported that starting prenatal or neonatal treatment early after seroconversion was protective against retinal disease. Finally, in the randomized TOXOGEST trial (Mandelbrot et al., 2018), early utero treatment with pyrimethamine + sulfadiazine was associated with a significantly lower incidence of cerebral ultrasound signs compared with spiramycin.

## 5 Discussion

Over the past fifty years, the age-specific seroprevalence to *T. gondii* has been constantly declining in Europe (Welton and Ades, 2005). The risk of infection with *T. gondii* thoughout life is much lower compared to the 1950s and 60s, as well as the risk of infection occurring during pregnancy.

Throughout the European studies the relatively low risk of clinical symptoms compared to the results from the United States, reported by the National Collaborative Chicago-Based, Congenital Toxoplasmosis Study (McLeod et al., 2006) fuelled controversies over whether this was due to improved management and treatment or simply a recruitment bias. In fact, the European cohorts were identified by systematic screening and therefore included congenitally infected children with no or few symptoms in contrast to the Chicago cohort that only included symptomatic children. Nonetheless, within the Lyon cohort (Wallon et al., 2013), the frequency of clinical signs of congenital toxoplasmosis among infected children at 3 years of age decreased between children born in the period 1996–2008 when prenatal screening and diagnosis with amniocentesis as well as early prenatal treatment were introduced, compared with the previous period 1987–1995 (46/1150 vs. 87/794; *p* = 0.012).

A review study looked at the cost/benefit of screening for congenital toxoplasmosis in a context of low prevalence as observed in the northern part of Europe and the USA, where the prevalence was 10% and the incidence during pregnancy was 0.05% in women at risk. The 1-year Incremental Cost-Effectiveness Ratio showed that prenatal screening would require investing €14,826 to avoid one adverse event (liveborn with CT, fetal loss, neonatal death or pregnancy termination) compared to neonatal screening. Extra investment increased cost up to €21,472 when considering a 15-year endpoint (Binquet et al., 2019). Other studies have found that screening programs are cost effective (Bobić et al., 2019; Prusa et al., 2017; Stillwaggon et al., 2011). Such conclusions can however be challenged because the models rely on estimations, some of which are imprecise. Data are missing from South America and Africa.

There is thus ongoing controversy regarding screening. The prenatal screening programs continue in Austria, France and Slovenia, as well as much of Italy, but has been stopped in Spain and Switzerland. Screening programs in Brazil are also to start. A decade ago, experts in the United Kingdom concluded against screening (Gilbert and Peckham, 2002), whereas other experts disagreed (Montoya, 2018) and a committee of the French College of Gynecology and Obstetrics recently concluded in favor of prenatal screening (Picone et al., 2020).

The screening programs in France and Austria have been in operation for 46 years with unchanged algorithms of diagnostics and treatment, despite a decline in seroprevalence and thus a declining risk to pregnant women. Prenatal screening programs aim at preventing maternal-fetal transmission of *T. gondii* in case of maternal seroconversion as well as preventing sequelae in infected infants (Wallon et al., 2013). In Europe, where genotype 2 is predominant, more than 75% of infections in newborns are asymptomatic, and there is a moderate level of proof that starting treatment as early as possible during pregnancy and up to a year after birth reduces the risk of retinochoroiditis later in life. Treatment tolerance of pyrimethamine-sulfadiazine with folinic acid is generally good. During prenatal treatment, the main risks are digestive intolerance, bone marrow suppression and allergies. Side effects are seen in infants (Schmidt et al., 2006) and treatment with pyrimethamine-sulfadoxine seems to be better tolerated (Teil et al., 2016).

It is by now well known that more pathogenic genotypes of *T. gondii* are found in South America and Africa, and the results from Europe cannot be used on other continents (Howe and Sibley, 1995; Galal et al., 2019; Hamidović et al., 2021). A comparison between ocular toxoplasmosis in Brazil and Europe clearly demonstrated the higher pathogenicity of human infections in Brazil (Gilbert et al., 2008). A higher proportion of children developed retinochoroiditis during the first year of life in Brazil than in Europe (15/30; 50% versus 29/281; 10%) and the risk of lesions by 4 years of age was much higher: the hazard ratio for Brazil versus Europe was 5.36 (95%CI: 3.17–9.08) (Gilbert et al., 2008). Another population-based study in the state of Minas Gerais, Southeastern Brazil, found that up to 80% of infected babies of untreated mothers were born with retinochoroiditis, with approximately 50% displaying active retinochoroiditis (Vasconcelos-Santos et al., 2009). Interestingly, peripheral circulation of viable parasites could also be demonstrated in the blood of some of these newborns (Carneiro et al., 2013). Amplification of *T. gondii* DNA in blood was possible in approximately 50%, being particularly associated with active retinochoroiditis (Costa et al., 2013). Whether these results mostly reflect increased pathogenicity of *T. gondii* in South America or lack of prenatal therapy remains to be determined. However, they enhance the argument for prenatal treatment, particularly in South America.

The consumption of raw and undercooked meat is a risk factor for infection. Over the past decades consumption of “ecological” meat i.e. products especially from pigs that has been kept partly outside of stables has become more popular as highlighted almost 20 years ago (Kijlstra et al., 2004). A recent study from Denmark found a 2% (95% CI = 0.4%–5%) seroprevalence of *T. gondii* in conventional finishers compared to 11% (95% CI = 6%–17%) in organic finishers (Olsen et al., 2020). The trends in production of free-range organically raised meat may indeed increase risk of *T. gondii* transmission from pigs to humas, as had been predicted by Jones and Dubey (2012). (Jones and Dubey, 2012).

One approach would be to move toward *Toxoplasma* free meat products. This is probably not possible for animals raised outdoor with exposure to the environment inherent to organic production.

What could replace systematic screening?

There is no algorithm to offer risk-based screening in the context of toxoplasmosis. Therefore, the alternatives are no screening or individualized decision-making based on women's preference. Home screening using saliva for detecting IgG-seroconversion is a possibility and the technology exists (Sampaio et al., 2014). Self-testing using saliva is attractive because it is non-invasive and easy to perform. Such self-testing at home is currently under consideration for COVID-19 (Hirst et al., 2021) There is at present no study or economic modeling on which to base such an approach for toxoplasmosis prevention.

Point of care tests exists based on blood samples and therefore does not provide the flexibility of home testing using saliva (Chapey et al., 2017; Gomez et al., 2018).

## 6 Conclusion

The current prenatal screening programs in Austria, France and Slovenia are based on screening algorithms developed in the 1950s and 60s when the epidemiological situation was quite different. The key question is if the programs prevent disease or if the funds used could be spend on other issues. The main argument for screening is to allow for timely treatment. However, periodic reappraisals of screening programs should be performed to make sure that they provide public health advantages. New screening methods that are simple, easy to use are urgently needed, new drugs should be developed and vaccines should be studied. Further clinical trials of therapies are urgently needed, especially in areas with a high burden of *T.gondii* infections and more pathogenic genotypes compared to Europe and North America, principally in South America and Africa.

## Declaration of Competing Interest

On behalf of all authors we declare no conflict of interest.
